# Association of Adiponectin rs1501299 and rs266729 Gene Polymorphisms With Nonalcoholic Fatty Liver Disease

**DOI:** 10.5812/hepatmon.9527

**Published:** 2013-05-21

**Authors:** Mohammad Hashemi, Hamideh Hanafi Bojd, Ebrahim Eskandari Nasab, Ali Bahari, Noor Allah Hashemzehi, Sara Shafieipour, Behzad Narouie, Mohsen Taheri, Saeid Ghavami

**Affiliations:** 1 Cellular and Molecular Research Center, Zahedan University of Medical Sciences, Zahedan, IR Iran; 2 Department of Clinical Biochemistry, School of Medicine, Zahedan University of Medical Sciences, Zahedan, IR Iran; 3 Department of Internal Medicine, School of Medicine, Mashhad University of Medical Sciences, Mashhad, IR Iran; 4 Department of Internal Medicine, School of Medicine, Zahedan University of Medical Sciences, Zahedan, IR Iran; 5 Department of Internal Medicine, School of Medicine, Kerman University of Medical Sciences, Kerman, IR Iran; 6 Genetic of Non-Communicable Diseases Research Center, School of Medicine, Zahedan University of Medical Sciences, Zahedan, IR Iran; 7 Department of Physiology, Manitoba Institute of Child Health, University of Manitoba, MB, Winnipeg, Canada

**Keywords:** Fatty Liver, Adiponectin, Polymorphism

## Abstract

**Background:**

Genetic and environmental factors are important for the development of nonalcoholic fatty liver disease (NAFLD). Adiponectin is a white and brown adipose tissue hormone, and have been found to play essential roles in the regulation of energy homoeostasis. Recent reports have identified a possible role of adiponectin in NAFLD via PPARγ pathway.

**Objectives:**

The present study was designed to find out the impact of adiponectin rs1501299 (276G/T) and rs266729 (-11377C/G) gene polymorphisms in NAFLD.

**Patients and Methods:**

Eighty-three patients with diagnosis of NAFLD, and 93 healthy subjects were included in the study. Tetra ARMS-PCR was designed to detect single nucleotide polymorphisms.

**Results:**

A significant difference was found between NAFLD and control group regarding the rs266729 polymorphism (χ2 = 7.35, P = 0.025). The rs266729 polymorphism increased the risk of NAFLD in codominant (CC vs. CG: OR = 2.18, 95% CI = 1.16 - 4.12, P = 0.016) and dominant (CC vs. CG/GG: OR = 2.31, 95% CI = 1.25 - 4.27; P = 0.008) inheritance tested models. The G allele increased the risk of NAFLD (OR = 1.63, 95% CI = 1.03 - 2.57, P = 0.037) in comparison with C allele. No significant difference was found between the groups concerning adiponectin rs1501299 gene polymorphism (χ2 = 0.70, P = 0.697).

**Conclusions:**

adiponectin rs266729 polymorphism might be a candidate gene, which determines the susceptibility to NAFLD. Larger studies are necessary to confirm these findings in various populations.

## 1. Background

Nonalcoholic fatty liver disease (NAFLD) is one of the most common forms of chronic liver diseases worldwide (1). NAFLD is an epidemic metabolic liver disease in many countries (2, 3). It is characterized by the association of hepatic steatosis with liver cell injury, lobular inflammation and variable fibrosis that could progress to cirrhosis (4). The prevalence of NAFLD is rising worldwide (5). The prevalence of NAFLD in Iranian children has found to be 7.1%, and significantly more common in older age group (6). The rate of NAFLD is strongly linked to obesity, insulin resistance and other components of the metabolic syndrome (7). NAFLD has been recognized as a leading cause of abnormal liver function tests. Its spectrum varies from simple fatty liver, which is usually a benign and nonprogressive condition, to nonalcoholic steatohepatitis (NASH), which may progress to cirrhosis (8, 9). In spite of the high prevalence of NAFLD, little is known about its pathogenesis. Recent studies suggest that both environmental and genetic factors are involved in the development and progression of NAFLD. Genetic risk factor for NAFLD may differ between different populations. Accordingly, replicating previously reported genetic associations in other populations (10-12) are desirable to determine the associations of the genetic risk in each population. Genetic polymorphisms of the glutathione S-transferase genes (GSTM1and GSTP1) (13), peroxisome proliferator-activated receptor gamma (PPARgamma) (14), liver fatty acid-binding protein (FABP1) (15), microsomal triglyceride transfer protein (MTTP) (16), leptin receptor gene (17), and adiponectin gene (11) are reported to be associated with NAFLD. Adiponectin is an adipose tissue–specific plasma protein, which is known to play important functions in modulating insulin sensitivity, glucose and energy homeostasis, glucose and lipid metabolism, and anti-inflammatory responses in the vascular system (18). Adiponectin is produced by adipocytes, and then secreted in the circulation of human healthy individuals at relatively high levels, which fluctuates between 5 and 30 μg/ml (19). However, the levels of this adipokine is reduced in patients with insulin resistance (20), type II diabetes mellitus (T2DM) (21, 22), obesity (23), cardiovascular disease (24), metabolic syndrome (25), and NAFLD (26-29).

The gene coding for adiponectin, is officially named ADIPOQ, located on chromosome 3q27, and consists of 3 exons and 2 introns, spanning a total of 16 kb of genomic sequence. Adiponectin contains 244 amino acids, a signal peptide, a collagen-like domain at its N-terminus and a globular domain at its C-terminus, which shares sequence similarities with collagens X and VIII, as well as the complement factor C1q (30, 31). There is growing evidence demonstrating the association of single nucleotide polymorphisms (SNPs) of the ADIPOQ gene with varying levels of circulating adiponectin. Two common SNPs, rs266729 ( −11377 C > G) and rs1501299 (+276 G > T) in the proximal promoter and intronic region of the ADIPOQ gene, respectively, have been widely studied by epidemiological studies. Variant alleles at rs266729, which is associated with lower adiponectin levels, has been shown to be related with obesity (32), body mass index (BMI) (33), type 2 diabetes (T2DM), diabetic nephropathy (34), and insulin sensitivity (35). Other variant, rs1501299, is correlated with decreased adiponectin expression, which might in turn lead to increased body weight and insulin resistance (36, 37). Although a few studies have examined the association between adiponectin gene polymorphisms with risk of NAFLD (10-12, 38, 39), but no study regarding the association of adiponectin variants and predisposition to NAFLD in an Iranian population has yet been published.

## 2. Objectives

The present study was aimed to evaluate the possible association between two adiponectin variants, 276 G/T (rs1501299) and –11377 C/G (rs266729), and susceptibility to NAFLD in a southeast Iranian population.

## 3. Materials and Methods

### 3.1. Study Groups

This case-control study was conducted in the Departments of Internal Medicine and Biochemistry at Zahedan University of Medical Sciences. The study groups consisted of 83 patients with NAFLD (50 men and 33 women; age 40.45 ± 12.12 years), and 93 healthy participants (42 men and 51 women; age 42.33 ± 16.25 years). The study design and the enrolment procedure have been previously described in detail (13, 16). NAFLD diagnosis was based on clinical symptoms, sonographic and laboratory findings. Patients with viral hepatitis B and C, autoimmune liver diseases, hemochromatosis, Wilson disease, alcohol intake of more than 100g/week, and chronic drug consumption were excluded from the study, which have been discussed previously (16, 40). Our healthy participants were carefully chosen from the same population who participated voluntarily in another study to investigate the prevalence of metabolic syndrome (41) and had normal findings for AST, ALT, blood pressure, waist circumference, blood glucose, body mass index, and normal lipid profile tests (16, 40). Blood samples were collected in Na-EDTA tubes from patients and healthy participants, and DNA were extracted from peripheral blood by salting-out method as described previously (42). In brief, 500 μL bloods was transferred to 1.5-mL microfuge tubes, and 1 mL cell lysis buffer (10 mM Tris-HCl, 11% w/v sucrose, 5 mM MgCl2, and 11% v/v Triton X-100) was added. Microfuge tubes were gently mixed and centrifuged for 2 min at 6000 rpm at room temperature, after which the supernatant was discarded. The procedure was repeated twice. Next, 300 μL buffer II (10 mM Tris-HCl, 10 mM EDTA, and 10 mM sodium citrate) and 40 μL 10% SDS were added, and the mixture incubated for 2 min at room temperature. Then, 100 μL saturated NaCl and 600 μL chloroform were added with gentle mixing, and the mixture centrifuged for 2 min at 6000 rpm. The supernatant was transferred to a new microfuge tube, where 700 μL cold isopropanol was added, followed by gentle mixing and centrifugation for 1 min at 12,000 rpm for 2 min at 4°C. The supernatant was discarded and 700 μL cold 70% ethanol was added. The suspension was gently mixed and centrifuged for 1 min at 12,000 rpm at 4°C. Pellets were subsequently dried before dissolving in 100 μL distilled water. The ethics committee of Zahedan University of Medical Sciences approved this study, and informed consent was obtained from all subjects.

### 3.2. Tetra Primer Amplification Refractory Mutation System PCR (tetra ARMS-PCR)

**Figure 1.**
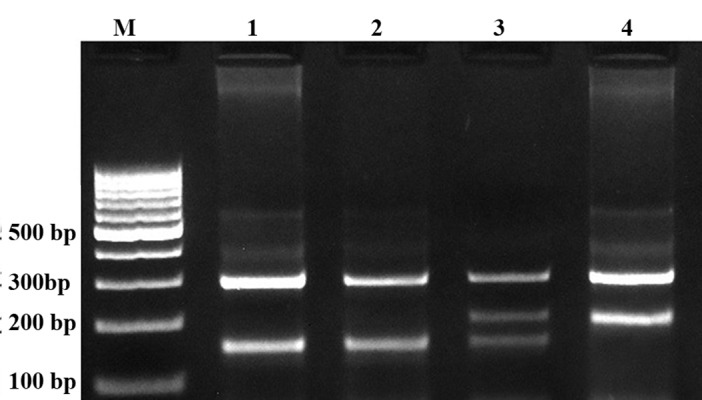
Representative PCR Products of T-ARMS-PCR Resolved by Agarose Gel Electrophoresis to Detect the Adiponectin rs266729 C/G Polymorphism The product sizes were 299 bp for control band, 155 bp for C allele, and 201 bp for G allele. M, DNA marker; lanes 1 and 2, CC; lane 3 CG; lane 4, GG.

**Figure 2.**
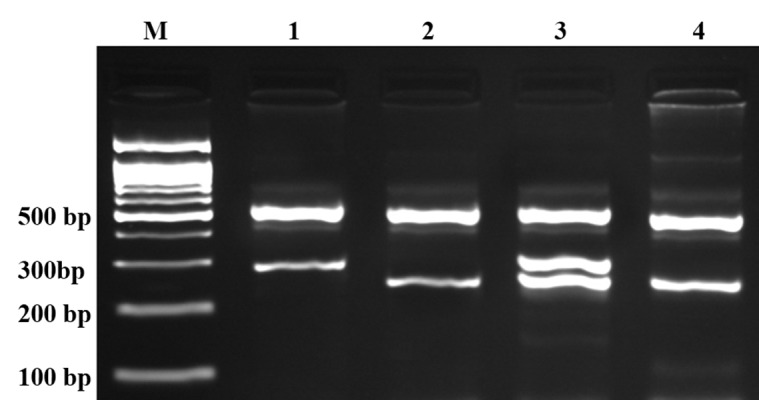
Electrophoresis Pattern of PCR Products of T-ARMS-PCR for Detection of Adiponectin rs1501299 G/T Polymorphism The product sizes were 244 bp for G allele, 292 bp for T allele and 476 bp for control band. M, DNA marker; lane 1, TT; lanes 2 and 4, GG; lane 3, GT.

In this study we designed a Tetra amplification refractory mutation system polymerase chain reaction (T-ARMS-PCR) for detection of polymorphisms of adiponectin. This method is simple, rapid and sensitive for the detection of single nucleotide polymorphism (43, 44). The adiponectin genomic sequence (NT_005612.16) was obtained from the National Center for Biotechnology Information (NCBI) (http://www.ncbi.nlm.nih.gov). The polymorphisms were searched, and primers for T-ARMS-PCR were designed. For adiponectin rs266729 polymorphism, we used two external primers (Forward outer: 5`- GGA CTG TGG AGA TGA TAT CTG GGG GGC A-3`, Reverse outer: 5`- TGG CCT AGA AGC AGC CTG GAG AAC TGG A-3`), and the two allele specific internal primers were (Forward inner (C allele): 5`- CTT GCA AGA ACC GGC TCA GAT CCT CCC-3`, Reverse inner (G allele): 5`- GAG CTG TTC TAC TGC TAT TAG CTC TGC-3`). Primers for the adiponectin rs1501299 polymorphism were as follows: Forward outer, 5`- GAG CTG TTC TAC TGC TAT TAG CTC TGC-3`; Reverse outer, 5`- GAA TAT GAA TGT ACT GGG AAT AGG GAT G-3`; Forward inner (G allele), 5`- CCT CCT ACA CTG ATA TAA ACT ATA TGA GGG-3`; Reverse inner (T allele), 5`- TGT GTC TAG GCC TTA GTT AAT AAT GAA CGA-3`. PCR reactions consisted of a total volume of 20 μL containing 250 μM dNTPs, 0.4 μM of each primer, 2 mM MgCl2, 1 U Taq DNA polymerase, and 50 ng genomic DNA. The PCR cycling conditions were 5 min at 95°C followed by 30 cycles of 30 s at 95°C, 27 s at 61°C for rs1501299, 30 s at 62°C for rs266729, and 25 s at 72°C for rs1501299, 27 s at 72°C for rs266729, with a final step at 72°C for 5 min to allow for complete extension of all PCR fragments. Each reaction was verified on a 2% agarose gel (Figures 1 and 2). The product sizes for rs1501299 polymorphism were 244-bp for G allele, 292-bp for T allele and 476-bp for two outer primers (control band). The product sizes for rs266729 were 299-bp for control band, 155-bp for C allele, and 201-bp for G allele. To certify genotyping quality, we regenotyped approximately 20% of the random samples, and found no genotyping errors.

### 3.3. Statistical Analysis

Statistical analysis was performed using commercial software (SPSS for Windows, V 18, SPSS Inc, Chicago, IL, USA). Genotype and allelic frequencies were compared between the groups by chi-squared test. Logistic regression analysis was applied to estimate odds ratio (OR) and 95% confidence intervals (CI) of genetic risk in NAFLD. A p value less than 0.05 was considered statistically significant.

## 4. Results

The genotype and allele frequencies of adiponectin -11377C/G (rs266729) gene polymorphisms are shown in Table 1. A significant difference was observed between the groups regarding rs266729 of adiponectin gene (χ ^2^ = 7.35, P = 0.025). The adiponectin rs266729 polymorphism increased the risk of NAFLD in codominant and dominant tested inheritance models (OR = 2.18, 95% CI = 1.16 – 4.12, P = 0.016, CC vs CG; and OR = 2.31, 95% CI = 1.25 - 4.27, P = 0.008, CC vs CG - GG, respectively). The minor allele frequency (MAF) (G allele) of rs266729 polymorphism in NAFLD and controls were 0.356 and 0.253, respectively. A significant difference was found between the groups concerning allele frequency (χ2 = 4.41, P = 0.037). The rs266729 G allele increased the risk of NAFLD in comparison with C allele (OR = 1.63, 95% CI = 1.03 - 2.57, P = 0.037). The adiponectin rs266729 polymorphism in controls was in HWE (χ ^2^ = 2.60, P = 0.111) but in cases was out of HWE (χ ^2^ = 12.86, P < 0.001).

**Table 1.** The Genotypes and Allele Distribution of Adiponectin rs266729 Gene Polymorphisms in Case (NAFLD) and Control Groups

rs266729 Polymorphism	Case, No. (%)	Control, No. (%)	OR^a^(95% CI)	P value
**Codominan**t				
CC	27 (32.5)	49 (52.7)	1.00	-
CG	53 (63.9)	41 (44.1)	2.18 (1.16-4.12)	0.016
GG	3 (3.6)	3 (3.2)	1.17 (0.31-9.33)	0.536
**Dominant**				
CC	27 (32.5)	49 (52.7)	1.00	-
CG+GG	56 (67.5)	44 (47.3)	2.31 (1.25-4.27)	0.008
**Recessive**				
CC+CG	80 (96.4)	90 (96.8)	1.00	-
GG	3 (3.6)	3 (3.2)	1.08 (0.21-5.63)	0.928
**Alleles**				
C	107 (64.4)	139 (74.7)	1.00	-
G	59 (35.6)	47 (25.3)	1.63 (1.03-2.57)	0.037

^a^Adjusted for gender and age

As shown in Table 2, there were no significant differences in the genotypes frequencies between the NAFLD and control groups regarding adiponectin rs1501299 (276G/T) polymorphism (χ ^2^ = 0.72, P = 0.697). The adiponectin rs1501299 polymorphism was not associated with NAFLD in codominant, dominant, and recessive tested inheritance models (Table 2). The MAF (T allele) of rs1501299 polymorphism in NAFLD and controls were 0.259 and 0.368, respectively. The allele frequency was not significant different between the groups (χ ^2^ = 0.46, P = 0.529). The rs1501299 polymorphism in cases and controls were in HWE (χ ^2^ = 2.65, P = 0.104, and χ ^2^ = 3.31, P = 0.070, respectively).

**Table 2.** The Genotypes and Allele Distribution of Adiponectin rs1501299 Gene Polymorphisms in Case (NAFLD) and Control Groups

rs1501299 polymorphism	Case, No. (%)	Control, No. (%)	OR^a^(95% CI)	P value
**Codominant**				
GG	42 (50.6)	53 (57.0)	1.00	-
GT	39 (47.0)	38 (40.8)	1.38 (0.75-2.56)	0.302
TT	2 (2.4)	2 (2.2)	1.11 (0.15-8.44)	0.919
**Dominant**				
GG	42 (50.6)	53 (57.0)	1.00	-
GT+TT	41 (49.4)	40 (43.0)	0.99 (0.97-1.01)	0.368
**Recessive**				
GG+TT	81 (97.6)	91 (97.8)		
TT	2 (2.4)	2 (2.2)	0.97 (0.13-7.21)	0.968
**Alleles**				
G	123 (74.1)	144 (63.2)	1.00	-
T	43 (25.9)	42 (36.8)	1.19 (0.73-1.94)	0.533

^a^adjusted for gender and age

## 5. Discussion

In the current study, we investigated the possible association between adiponectin gene polymorphisms and NAFLD in a sample of Iranian population in the southeast of Iran. We found that rs266729 (-11377 G/C) polymorphism increased the risk of NAFLD while there was no association between rs1501299 (+276 G/T) polymorphism and NAFLD in the population. Recently, a growing number of studies have evaluated the impact of adiponectin gene polymorphisms on NAFLD in different populations (10-12, 38, 39). Gupta et al. (10) have investigated two functional polymorphisms of adiponectin gene (-11377 G/C and +45 T/G) in NAFLD, and found an association between these genetic polymorphisms and adiponectin levels and severity of NAFLD in an Indian population. In accordance with our findings, they found that homozygous mutant genotype of adiponectin variant -11377 C/G was significantly more prevalent in patients with NAFLD than in controls, and that the presence of 'G' allele at position -11377 C/G was associated with necroinflammatory grade and reduced adiponectin levels. In another study by the same research team (45), it was shown that the adiponectin rs1501299 (+276 G/T) polymorphism was associated with increasing body mass index (BMI), waist-hip ratio (WHR), and systolic blood pressure (SBP), the main quantitative traits of T2DM. Besides, Musso et al (11) and Zhou et al. (12) have found an association between +45 T/G and +276 G/T polymorphisms of adiponectin gene and risk of NAFLD in their studies. However, Wong et al have found no association between genetic polymorphism of adiponectin at positions 11391, -11377, +45, and +276 and NAFLD in Chinese patients (38). Although adiponectin +276 G/T polymorphism was not significantly different between NAFLD and controls, but among females, the GG genotype was reported to be significantly more prevalent in patients with NAFLD (39). Considering other adiponectin polymorphisms, Tokushige et al. have found no association between +45 G/T polymorphism and NAFLD. In their study the frequency of +45 GG genotype was significantly higher in the severe fibrosis group compared to the mild fibrosis group (39). Within the body’s system, the liver plays a vital task in regulating fatty acid and triglyceride (TG) metabolism by synthesizing, storing, releasing and oxidizing free fatty acids (FFA). Any disharmony in the pathways involved in triacylglycerol release, synthesis or oxidation could contribute to its accumulation in the liver (46). NAFLD is the most common reason for abnormal liver function, and may occur in 10-30% of the population (47). Amassment of triglycerides inside hepatocytes, chronic oxidative stress levels, insulin resistance, inflammation and fibrosis in combination make NAFLD a complicated disease (48). NAFLD can progress from simple steatosis to NASH, hepatocyte necrosis, fibrosis, and cirrhosis of the liver (49). The major risk factors for NAFLD are glucose intolerance and T2DM, obesity, metabolic syndrome and dyslipidaemia (50). Accumulating evidence from animal and human studies has proposed that adiponectin regulates hepatic and peripheral glucose and lipid metabolism (19, 51-53). In the liver, adiponectin decreases hepatic glucose production and reduces free fatty acid turnover, so that blood levels of adiponectin are negatively correlated with triglyceride (TG), and positively with low-density lipoprotein particle size and high-density lipoprotein cholesterol (HDL-C) levels (54). Furthermore, mRNA levels of adiponectin and plasma adiponectin are reduced in adipose tissue of patients with obesity and T2DM or coronary artery disease, suggesting that hypoadiponectinemia may contribute to the pathogenesis of the development of NAFLD from steatosis to steatohepatitis (55). The augment of adiponectin levels following a fat meal is thought to be an acute adaptive mechanism increasing removal of FFA and catabolism of triglyceride-rich lipoprotein. This compensatory mechanism is regulated by genetic factors and is jeopardized to a higher extent once inappropriate dietary habits are superimposed on an unfavorable genetic background. In this condition, adipocytes lose their “compensatory” ability to acutely release adiponectin in response to a fat load. The loss of this “metabolic flexibility” would be an early sign of adipocyte dysfunction and would result in excessive postprandial lipemia, enhanced FFA, and lipid uptake by the liver and adipose tissue, (56, 57) ultimately leading to NAFLD, visceral obesity, and lower fasting adiponectin levels. All together population-based studies on functional polymorphisms of adiponectin have revealed that polymorphisms in adiponectin is highly variable in different populations and its dependence to NAFLD risk and severity is environmental and population depended. Our study showed that adiponectin rs266729 polymorphism might be a candidate gene, which determines the susceptibility to NAFLD in a southeast Iranian population. One limitation of this study is its relatively small sample size. Therefore, the results need to be interpreted with caution. Larger studies with different ethnicities are necessary to confirm our findings in various populations.
